# Crystallographic and spectroscopic characterization of 2-bromo-*p*-tolu­aldehyde

**DOI:** 10.1107/S2056989025006127

**Published:** 2025-07-29

**Authors:** Eden E. Lanham, Joseph M. Tanski

**Affiliations:** a Department of Chemistry Vassar College,Poughkeepsie NY 12604 USA; Texas A & M University, USA

**Keywords:** crystal structure, π-stacking, benzaldehyde derivative

## Abstract

The title compound (systematic name: 2-bromo-4-methyl­benzaldehyde), C_8_H_7_BrO, is a halogenated benzaldehyde derivative. The mol­ecule contains an aldehyde moiety, *ortho* bromine, and *para* methyl group. Packing *via* van der Waals forces, the mol­ecules are arranged with both offset face-to-face and an edge-to-face π-stacking inter­action revealed by Hirshfeld surface characterization.

## Abstract 2

The mol­ecule of the title halogenated benzaldehyde derivative contains an aldehyde moiety, *ortho* bromine, and *para* methyl group. Packing *via* van der Waals forces, the mol­ecules are arranged with both offset face-to-face and an edge-to-face π-stacking inter­action revealed by Hirshfeld surface characterization.

## 1. Chemical context

Benzaldehydes are a class of mol­ecules commonly used as key ingredients in many natural fruit flavorings (Jabba *et al.*, 2020 ▸; Kosmider *et al.*, 2016 ▸). Benzaldehydes exhibit a wide range of properties; some of their derivatives have been investigated as potential carcinogens, particularly as flavor ingredients commonly found in e-cigarettes (Jabba *et al.*, 2020 ▸). Conversely, others have been researched for their anti­cancer properties (Saitoh & Saya, 2016 ▸; Takeuchi *et al.*, 1978 ▸). The title compound, 2-bromo-4-methyl­benzaldehyde (I), more widely known as 2-bromo-*p*-tolu­aldehyde, is a benzaldehyde derivative with a bromine and methyl group. This compound may be synthesized by converting 2-bromo-4-methyl­aniline to the aldehyde *via* the benzene­diazo­nium chloride (Jolad & Rajagopalan, 1966 ▸). The title compound has seen recent applications in the synthesis of non-linear optical materials (Rahman *et al.*, 2025 ▸), as the starting material in the preparation of a protein kinase inhibitor (Defois *et al.*, 2024 ▸), and in ruthenium-catalyzed aldehyde annulation to prepare indenes (Ma *et al.*, 2025 ▸).
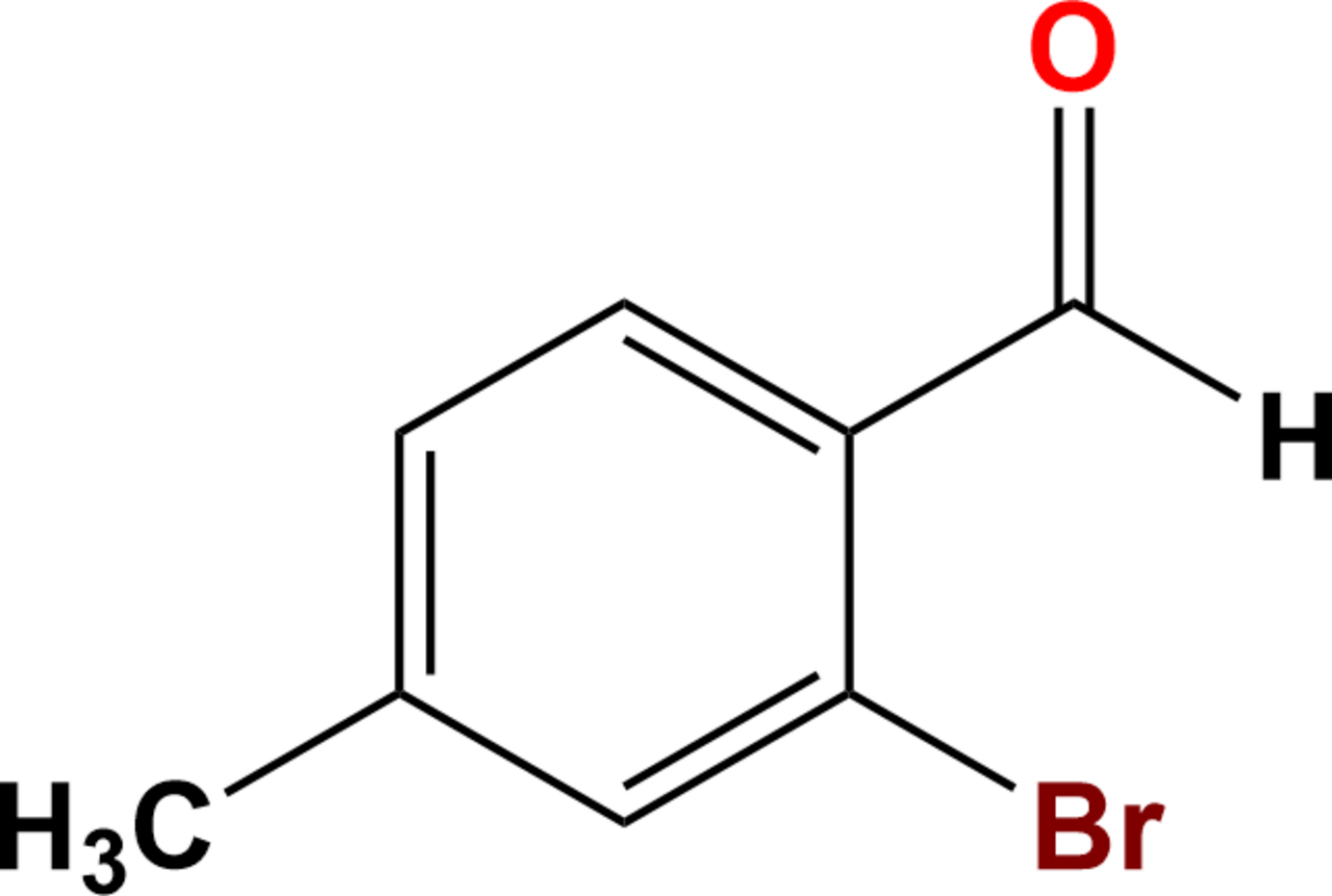


## 2. Structural commentary

**Figure 1:**
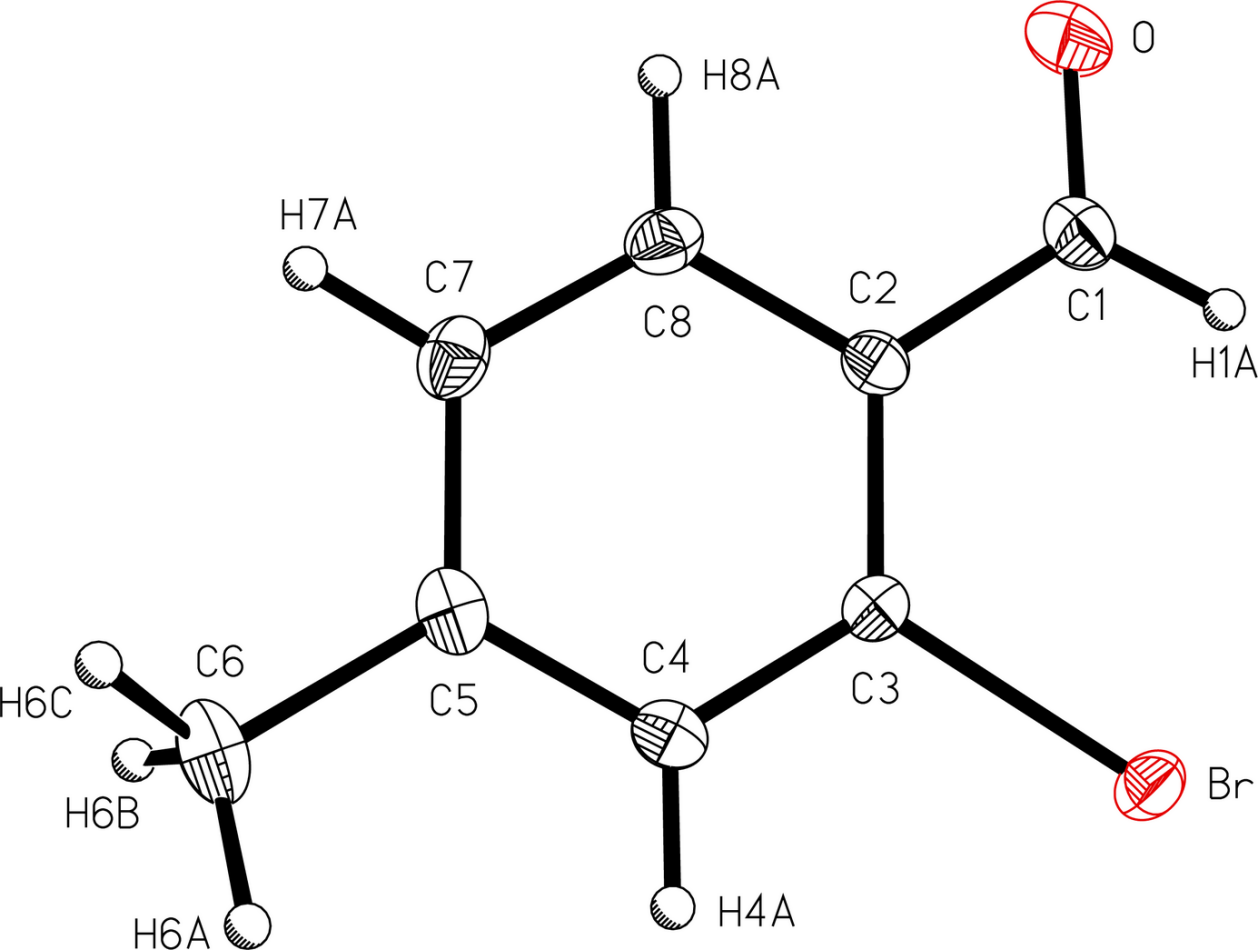
A view of 2-bromo-*p*-tolu­aldehyde (I) with the atom-numbering scheme. Displacement ellipsoids are shown at the 50% probability level.

The mol­ecular structure of the title compound (Fig. 1 ▸) shows the aldehyde group located at the *para* position relative to the methyl group on the aromatic ring. The aldehyde is oriented with the oxygen atom rotated opposite an *ortho* bromine atom to avoid electronic repulsion, resulting in an intra­molecular Br⋯H1*A* inter­action at 2.7895 (14) Å and an angle between the aldehyde group and the plane of the aromatic ring of 10.60 (13)°.

## 3. Supra­molecular features

**Figure 2:**
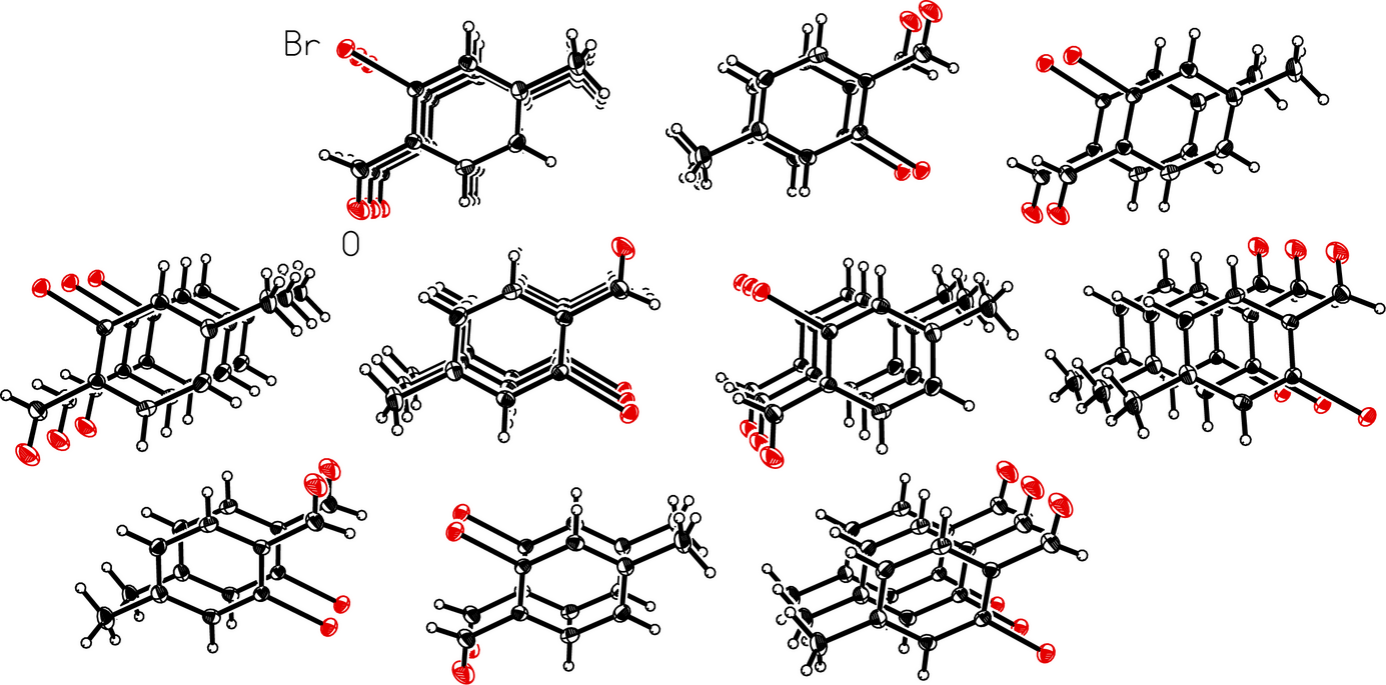
A view of the mol­ecular packing in 2-bromo-*p*-tolu­aldehyde (I). Displacement ellipsoids are shown at the 50% probability level.

**Figure 3:**
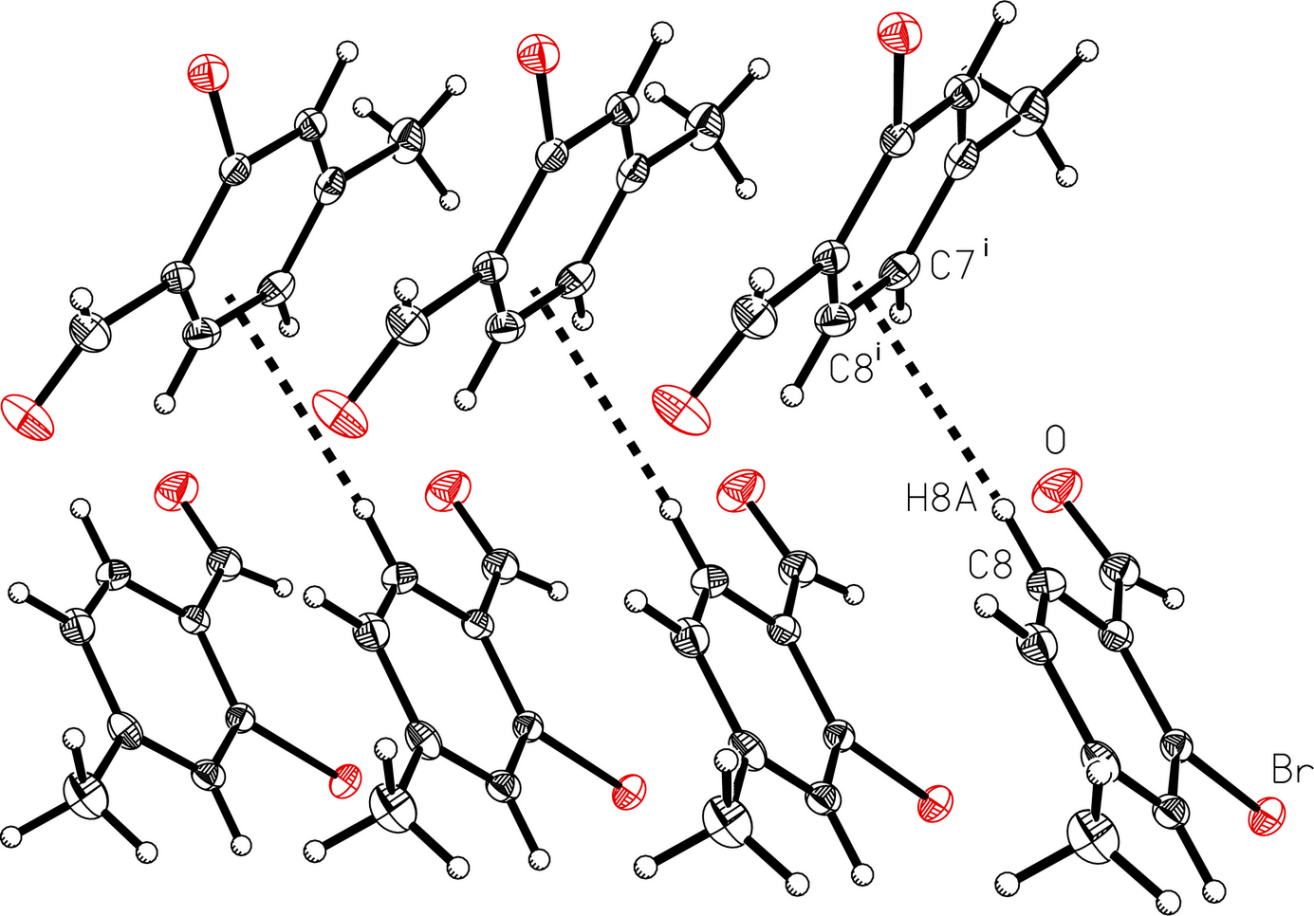
A view of the face-to-face and edge-to-face (C8—H8*A*⋯π^i^ shown *via* dashed lines) π-stacking geometrical arrangement in 2-bromo-*p*-tolu­aldehyde (I). Displacement ellipsoids are shown at the 50% probability level. Symmetry code: (i) 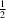 − *x*, −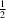 + *y*, 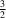 − *z*.

**Figure 4:**
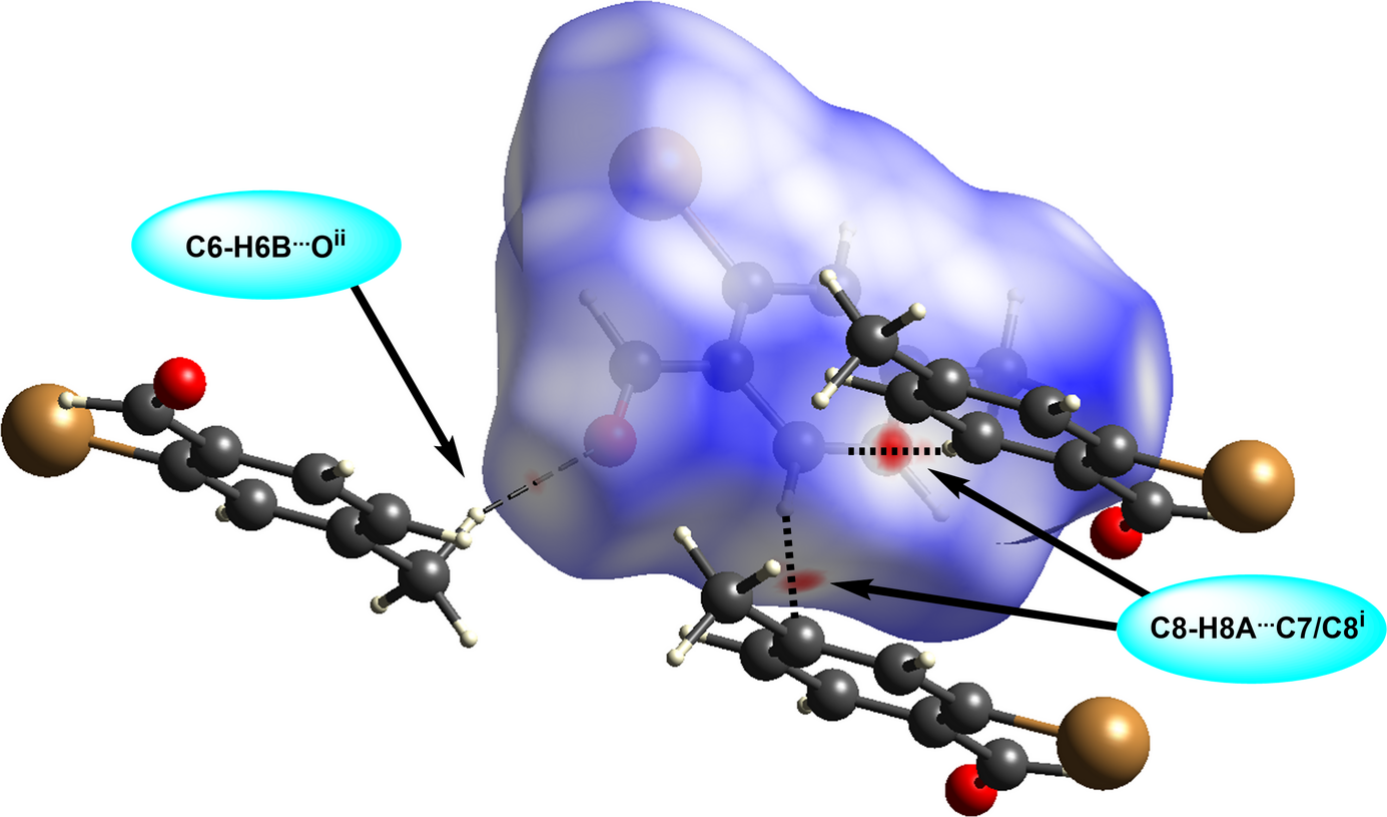
Hirshfeld surface of 2-bromo-*p*-tolu­aldehyde (I) mapped over *d*_norm_ showing *via* dashed lines the C8—H8*A*⋯π^i^ and C6—H6*B*⋯O^ii^ inter­actions. Symmetry codes: (i) 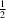 − *x*, −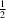 + *y*, 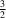 − *z*; (ii) −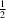 + *x*, 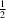 − *y*, −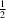 + *z*.

**Figure 5:**
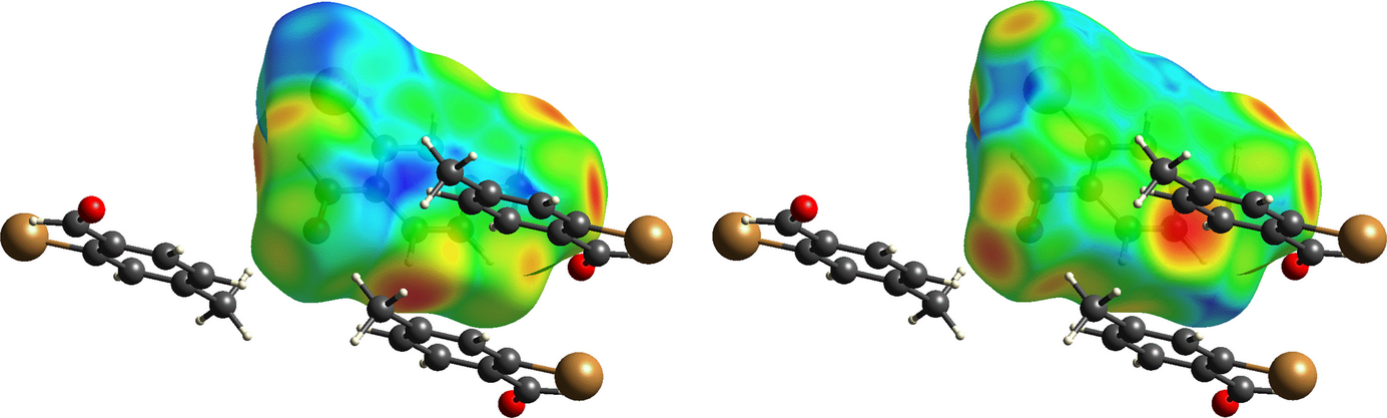
Hirshfeld surface of 2-bromo-*p*-tolu­aldehyde (I) mapped over *d*_i_ (left) and *d*_e_ (right) showing the C8—H8*A*⋯π^i^ inter­action.

**Figure 6:**
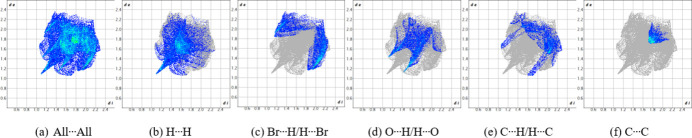
The (*a*) full two-dimensional fingerprint plot for 2-bromo-*p*-tolu­aldehyde (I) and individual fingerprint plots for (*b*) H⋯H (34.6%), (*c*) Br⋯H/H⋯Br (20.4%), (*d*) O⋯H/H⋯O (17.1%), (*e*) C⋯H/H⋯C (13.0%) and (*f*) C⋯C (6.7%) contacts.

The title compound packs together in the solid state *via* van der Waals forces (Fig. 2 ▸). The shortest distance between bromine atoms in adjacent mol­ecules is 3.9641 (3) Å, which exceeds the sum of the van der Waals radii of bromine (3.70 Å; (Bondi, 1964 ▸). In an offset head-to-tail stacking motif running parallel to the crystallographic *b*-axis, the mol­ecules of 2-bromo-*p*-tolu­aldehyde are arranged with the polar aldehyde and bromine groups in proximity to each other with a Br⋯H_aldehyde_ inter­action, Br⋯H1*A*, with H–acceptor distance of 3.0651 (14) Å, longer than the observed intra­molecular Br⋯H inter­action. The stacks further pack in an offset fashion to maximize hydro­phobic-like inter­actions of the non-polar tolyl groups. Both face-to-face and edge-to-face π-stacking geometrical arrangement of the aromatic rings are apparent, although both are highly offset (Fig. 3 ▸). The face-to-face π-stacking arrangement is characterized by a ring centroid-to-centroid distance of 3.9641 (3) Å, centroid-to-plane distance of 3.410 (1) Å, and ring-offset slippage parameter of 2.021 (2) Å. The edge-to-face π-stacking arrangement is revealed by the Hirshfeld surface calculated with *CrystalExplorer21* (Spackman *et al.*, 2021 ▸), mapped over *d*_norm_ in the range −0.0622 to 1.0811 a.u. (Fig. 4 ▸). The brighter red spot on the right of the surface indicates the offset edge-to-face C—H⋯π inter­action of the C8—H8*A* proton directed towards the C7–C8 edge of the mol­ecule within the surface, with an H8*A*⋯C7–C8 bond centroid distance of 2.770 Å. The red spot at the bottom of the surface represents the equivalent inter­action originating from the mol­ecule within the surface. The directionality of the C—H⋯π inter­action is confirmed when the surface is mapped over *d_e_* in the range 1.0656 to 2.3698 a.u. and *d*_i_ in the range 1.0646 to 2.3113 a.u. (Fig. 5 ▸), with a bright-red spot on the right of the surface when mapped over *d*_e_ indicating a short contact from the surface to the atom outside, and conversely a bright-red spot on the bottom of the surface when mapped over *d*_i_ indicating a short contact from the surface to the atom inside. The less intense red spot on the left of the *d*_norm_ surface indicates a weak C—H⋯O inter­action, C6—H6*B*⋯O, with an H–acceptor distance of 2.655 (10) Å. The two-dimensional fingerprint plots (Fig. 6 ▸) show that no single inter­action dominates, the most important inter­atomic contacts, summing to 91.8%, being (*b*) H⋯H (34.6%), (*c*) Br⋯H/H⋯Br (20.4%), (*d*) O⋯H/H⋯O (17.1%), (*e*) C⋯H/H⋯C (13.0%) and (*f*) C⋯C (6.7%) contacts. Additionally, Br contacts with C, Br and O together contribute 5.7%, and with the contribution of 2.4% O⋯C contacts, the total reaches 99.9%.

## 4. Database survey

The Cambridge Structural Database (version 6.00, April 2025; Groom *et al.*, 2016 ▸) contains related aromatic bromo­benzaldehydes. 4-Bromo­benzaldehyde (CSD Refcode YICFEV01; Ndima *et al.*, 2021 ▸) crystallizes with two mol­ecules in the asymmetric unit and features C—Br bond lengths of 1.891 and 1.895 Å, similar to the title compound at 1.9040 (13) Å, whereas the smaller angle between the aldehyde group and the plane of the aromatic rings of 0.85 and 2.07° in the related structure emphasizes in the impact of the *ortho* bromine regiochemistry in the title compound, which twists the aldehyde 10.60 (13)° out of the plane of the aromatic ring. Similarly to the title compound, related structure 1-bromo-2-naphthaldehyde (Refcode FADWIQ; Koppenhoefer & Bats, 1986 ▸) also has the aldehyde oriented with the oxygen atom rotated opposite the *ortho* bromine atom resulting in an intra­molecular Br⋯H inter­action at 2.653 Å, although the aldehyde carbonyl appears to be fully conjugated to the naphthyl, displaying an angle between the aldehyde group and the plane of the aromatic ring of 0°.

## 5. Synthesis and crystallization

2-Bromo-*p*-tolu­aldehyde (technical grade) was purchased from Aldrich Chemical Company, USA, and was used as received.

## 6. Refinement

Crystal data, data collection and structure refinement details are summarized in Table 1 ▸. All non-hydrogen atoms were refined anisotropically. Hydrogen atoms on carbon were included in calculated positions and refined using a riding model with: C—H = 0.95, 0.95 and 0.98 Å and *U*_iso_(H) = 1.2, 1.2 and 1.5 × *U*_eq_(C) of the aryl, aldehyde and methyl C-atoms, respectively.

## 7. Analytical Data

^1^H NMR (Bruker Avance III HD 400 MHz, CDCl_3_): δ 2.4 (*s*, 3H, C*H*_3_), δ 7.23 (*dd*, 1H, C_ar­yl_*H, J_ortho_ =* 7.8 Hz, *J_meta_ =* 0.8 Hz), δ 7.46 (*s*, 1H, C_ar­yl_*H*), δ 7.82 (*d*, 1H, C_ar­yl_*H, J_ortho_ =* 7.8 Hz), δ 10.32 (*s*, 1H, C*H*O). ^13^C NMR (^13^C{^1^H}, 100.6 MHz, CDCl_3_): δ 20.2 (*C*H_3_), δ 127.0 (*C*_ar­yl_), δ 128.9 (*C*_ar­yl_H), δ 129.9 (*C*_ar­yl_H), δ 131.2 (*C*_ar­yl_), δ 134.1 (*C*_ar­yl_H), δ 146.7 (*C*_ar­yl_), δ 190.2 (*C*HO). IR (Thermo Nicolet iS50, ATR, cm^−1^): 3029 (*w*, C_ar­yl_H str), 2953 (*w*, C_alk­yl_H str), 2923 (*w*, C_alk­yl_H str), 2858 (*m*) and 2752 (*w*) (CHO Fermi doublet), 1683 (*s*, CO str), 1648 (*m*), 1598 (*s*), 1560 (*m*), 1499 (*w*), 1482 (*m*), 1445 (*m*), 1380 (*s*), 1292 (*w*), 1267 (*s*), 1207 (*s*), 1141 (*m*), 1038 (*s*), 998 (*m*), 873 (*m*), 864 (*s*), 818 (*s*), 780 (*s*), 693 (*s*), 672 (*m*), 611 (*s*), 532 (*m*), 440 (*s*), 434 (*s*). GC/MS (Hewlett-Packard MS 5975/GC 7890): *M*+ = 199 amu.

## Supplementary Material

Crystal structure: contains datablock(s) global, I. DOI: 10.1107/S2056989025006127/jy2061sup1.cif

Structure factors: contains datablock(s) I. DOI: 10.1107/S2056989025006127/jy2061Isup2.hkl

Supporting information file. DOI: 10.1107/S2056989025006127/jy2061Isup3.cml

CCDC reference: 2471403

Additional supporting information:  crystallographic information; 3D view; checkCIF report

## Figures and Tables

**Table 1:** Experimental details

Crystal data	
Chemical formula	C_8_H_7_BrO
*M*_r_	199.05
Crystal system, space group	Monoclinic, *P*2_1_/*n*
Temperature (K)	125
*a*, *b*, *c* (Å)	11.4432 (8), 3.9641 (3), 16.8225 (11)
β (°)	102.838 (1)
*V* (Å^3^)	744.03 (9)
*Z*	4
Radiation type	Mo *K*α
μ (mm^−1^)	5.45
Crystal size (mm)	0.38 × 0.33 × 0.25

Data collection	
Diffractometer	Bruker APEXII CCD
Absorption correction	Multi-scan (*SADABS*; Krause *et al.*, 2015 ▸)
*T*_min_, *T*_max_	0.26, 0.34
No. of measured, independent and observed [*I* > 2σ(*I*)] reflections	17211, 2269, 2159
*R*_int_	0.023
(sin θ/λ)_max_ (Å^−1^)	0.714

Refinement	
*R*[*F*^2^ > 2σ(*F*^2^)], *wR*(*F*^2^), *S*	0.017, 0.045, 1.08
No. of reflections	2269
No. of parameters	93
H-atom treatment	H-atom parameters constrained
Δρ_max_, Δρ_min_ (e Å^−3^)	0.43, −0.34

Computer programs: *Bruker Instrument Service*, *APEX3* and *SAINT* (Bruker, 2013 ▸), *SHELXT2018/2* (Sheldrick, 2015*a* ▸), *SHELXL2017/1* (Sheldrick, 2015*b* ▸), *SHELXTL2014* (Sheldrick, 2008 ▸), *OLEX2* (Dolomanov *et al.*, 2009 ▸), and *Mercury* (Macrae *et al.*, 2020 ▸).

## supplementary crystallographic information

### 2-Bromo-4-methylbenzaldehyde Crystal data

**Table:**

C_8_H_7_BrO	*F*(000) = 392
*M**_r_* = 199.05	*D*_x_ = 1.777 Mg m^−^^3^
Monoclinic, *P*2_1_/*n*	Mo *K*α radiation, λ = 0.71073 Å
*a* = 11.4432 (8) Å	Cell parameters from 9969 reflections
*b* = 3.9641 (3) Å	θ = 2.4–30.5°
*c* = 16.8225 (11) Å	µ = 5.45 mm^−^^1^
β = 102.838 (1)°	*T* = 125 K
*V* = 744.03 (9) Å^3^	Plate, colourless
*Z* = 4	0.38 × 0.33 × 0.25 mm

### 2-Bromo-4-methylbenzaldehyde Data collection

**Table:**

Bruker APEXII CCD diffractometer	2269 independent reflections
Radiation source: sealed X-ray tube, Bruker APEX-II CCD	2159 reflections with *I* > 2σ(*I*)
Graphite monochromator	*R*_int_ = 0.023
Detector resolution: 8.3333 pixels mm^-1^	θ_max_ = 30.5°, θ_min_ = 2.0°
φ and ω scans	*h* = −16→16
Absorption correction: multi-scan (SADABS; Krause *et al.*, 2015)	*k* = −5→5
*T*_min_ = 0.26, *T*_max_ = 0.34	*l* = −23→24
17211 measured reflections	

### 2-Bromo-4-methylbenzaldehyde Refinement

**Table:**

Refinement on *F*^2^	Secondary atom site location: difference Fourier map
Least-squares matrix: full	Hydrogen site location: inferred from neighbouring sites
*R*[*F*^2^ > 2σ(*F*^2^)] = 0.017	H-atom parameters constrained
*wR*(*F*^2^) = 0.045	*w* = 1/[σ^2^(*F*_o_^2^) + (0.0209*P*)^2^ + 0.4808*P*] where *P* = (*F*_o_^2^ + 2*F*_c_^2^)/3
*S* = 1.08	(Δ/σ)_max_ = 0.003
2269 reflections	Δρ_max_ = 0.43 e Å^−^^3^
93 parameters	Δρ_min_ = −0.34 e Å^−^^3^
0 restraints	Extinction correction: *SHELXL2017/1* (Sheldrick 2015b)
Primary atom site location: dual	Extinction coefficient: 0.0085 (7)

### 2-Bromo-4-methylbenzaldehyde Special details

**Table:**

Geometry. All esds (except the esd in the dihedral angle between two l.s. planes) are estimated using the full covariance matrix. The cell esds are taken into account individually in the estimation of esds in distances, angles and torsion angles; correlations between esds in cell parameters are only used when they are defined by crystal symmetry. An approximate (isotropic) treatment of cell esds is used for estimating esds involving l.s. planes.

### 2-Bromo-4-methylbenzaldehyde Fractional atomic coordinates and isotropic or equivalent isotropic displacement parameters (Å^2^)

**Table:**

	*x*	*y*	*z*	*U*_iso_*/*U*_eq_	
Br	0.61962 (2)	0.86694 (3)	0.61166 (2)	0.01803 (5)	
O	0.28890 (10)	0.2568 (3)	0.56866 (7)	0.0305 (2)	
C1	0.37283 (12)	0.4513 (4)	0.58656 (8)	0.0204 (3)	
H1A	0.40198	0.554074	0.543759	0.024*	
C2	0.43181 (11)	0.5358 (3)	0.67170 (7)	0.0147 (2)	
C3	0.53957 (11)	0.7144 (3)	0.69284 (8)	0.0138 (2)	
C4	0.59307 (11)	0.7850 (3)	0.77369 (8)	0.0159 (2)	
H4A	0.666864	0.904404	0.78654	0.019*	
C5	0.53869 (12)	0.6811 (3)	0.83594 (8)	0.0173 (2)	
C6	0.59545 (14)	0.7624 (4)	0.92352 (8)	0.0246 (3)	
H6A	0.652269	0.948864	0.925516	0.037*	
H6B	0.637915	0.563344	0.949925	0.037*	
H6C	0.533058	0.827998	0.952079	0.037*	
C7	0.43039 (12)	0.5029 (3)	0.81548 (8)	0.0188 (2)	
H7A	0.392109	0.431425	0.857244	0.023*	
C8	0.37879 (11)	0.4303 (3)	0.73479 (8)	0.0180 (2)	
H8A	0.305944	0.306442	0.722046	0.022*	

### 2-Bromo-4-methylbenzaldehyde Atomic displacement parameters (Å^2^)

**Table:**

	*U*^11^	*U*^22^	*U*^33^	*U*^12^	*U*^13^	*U*^23^
Br	0.01877 (7)	0.01856 (7)	0.01827 (7)	−0.00280 (4)	0.00737 (5)	0.00088 (4)
O	0.0269 (5)	0.0362 (6)	0.0245 (5)	−0.0129 (5)	−0.0029 (4)	−0.0015 (5)
C1	0.0202 (6)	0.0224 (6)	0.0171 (6)	−0.0019 (5)	0.0008 (5)	−0.0002 (5)
C2	0.0132 (5)	0.0148 (5)	0.0153 (5)	0.0013 (4)	0.0018 (4)	−0.0001 (4)
C3	0.0139 (5)	0.0127 (5)	0.0155 (5)	0.0013 (4)	0.0047 (4)	0.0000 (4)
C4	0.0144 (5)	0.0147 (5)	0.0176 (6)	0.0014 (4)	0.0016 (4)	−0.0014 (4)
C5	0.0201 (6)	0.0161 (5)	0.0153 (5)	0.0061 (4)	0.0028 (4)	−0.0007 (4)
C6	0.0315 (7)	0.0266 (7)	0.0145 (6)	0.0045 (6)	0.0024 (5)	−0.0029 (5)
C7	0.0192 (6)	0.0201 (6)	0.0189 (6)	0.0044 (5)	0.0082 (5)	0.0028 (5)
C8	0.0141 (5)	0.0191 (6)	0.0216 (6)	0.0004 (4)	0.0056 (4)	0.0013 (5)

### 2-Bromo-4-methylbenzaldehyde Geometric parameters (Å, º)

**Table:**

Br—C3	1.9040 (13)	C5—C7	1.4013 (19)
O—C1	1.2162 (18)	C5—C6	1.5071 (19)
C1—C2	1.4796 (18)	C6—H6A	0.98
C1—H1A	0.95	C6—H6B	0.98
C2—C3	1.3977 (17)	C6—H6C	0.98
C2—C8	1.3984 (18)	C7—C8	1.3849 (19)
C3—C4	1.3904 (17)	C7—H7A	0.95
C4—C5	1.3941 (19)	C8—H8A	0.95
C4—H4A	0.95		
			
O—C1—C2	123.27 (13)	C7—C5—C6	120.89 (13)
O—C1—H1A	118.4	C5—C6—H6A	109.5
C2—C1—H1A	118.4	C5—C6—H6B	109.5
C3—C2—C8	117.74 (11)	H6A—C6—H6B	109.5
C3—C2—C1	123.22 (12)	C5—C6—H6C	109.5
C8—C2—C1	119.04 (12)	H6A—C6—H6C	109.5
C4—C3—C2	121.51 (12)	H6B—C6—H6C	109.5
C4—C3—Br	117.40 (9)	C8—C7—C5	120.44 (12)
C2—C3—Br	121.08 (9)	C8—C7—H7A	119.8
C3—C4—C5	120.15 (12)	C5—C7—H7A	119.8
C3—C4—H4A	119.9	C7—C8—C2	121.29 (12)
C5—C4—H4A	119.9	C7—C8—H8A	119.4
C4—C5—C7	118.85 (12)	C2—C8—H8A	119.4
C4—C5—C6	120.25 (13)		
			
O—C1—C2—C3	169.24 (14)	C3—C4—C5—C7	0.54 (19)
O—C1—C2—C8	−10.0 (2)	C3—C4—C5—C6	−178.96 (12)
C8—C2—C3—C4	0.20 (18)	C4—C5—C7—C8	0.30 (19)
C1—C2—C3—C4	−179.10 (12)	C6—C5—C7—C8	179.80 (13)
C8—C2—C3—Br	179.59 (9)	C5—C7—C8—C2	−0.9 (2)
C1—C2—C3—Br	0.29 (17)	C3—C2—C8—C7	0.65 (19)
C2—C3—C4—C5	−0.80 (19)	C1—C2—C8—C7	179.99 (12)
Br—C3—C4—C5	179.79 (9)		

### 2-Bromo-4-methylbenzaldehyde Hydrogen-bond geometry (Å, º)

**Table:**

*D*—H···*A*	*D*—H	H···*A*	*D*···*A*	*D*—H···*A*
C1—H1*A*···Br^i^	0.95	3.07	3.5842 (14)	116

Symmetry code: (i) −*x*+1, −*y*+1, −*z*+1.
